# Mussel-inspired coaxial electrospun nanofiber membranes promote scarless oral mucosal repair by enhancing macrophage M2 polarization

**DOI:** 10.1016/j.mtbio.2026.103272

**Published:** 2026-05-30

**Authors:** Junpeng Chen, Jinpeng Jiang, Wenyi Shen, Bingjie Xu, Haina Miao, Yangxi Cheng, Yi Zheng, Huiyong Zhu, Dan Yu

**Affiliations:** a Department of Oral and Maxillofacial Surgery, The First Affiliated Hospital, Zhejiang University School of Medicine, Hangzhou, China; b Zhejiang University School of Medicine, Hangzhou, China; c Department of Stomatology, Zhongshan City People's Hospital, Zhongshan, Guangdong Province, China; d School of Materials Science and Engineering, Northwestern Polytechnical University, Xi'an, China

**Keywords:** Coaxial electrospinning, Oral mucosal repair, Macrophage polarization, PPARγ, Dopamine

## Abstract

The repair of oral mucosal defects is often hindered by the failure of traditional materials to maintain adhesion in humid, dynamic environments. Inspired by the wet-adhesion properties of mussels, we developed a biomimetic self-adhesive nanofibrous membrane via coaxial electrospinning. This membrane utilizes a polycaprolactone (PCL) core for mechanical strength and a dopamine (DA)-modified gelatin methacrylate (GelMA) shell for biocompatibility and adhesion. Experimental results demonstrate that the membrane possesses excellent mechanical properties, with a tensile strength of 2.52 MPa and a swelling degree of 554.6%. The incorporation of 2% DA significantly enhanced wet-adhesive strength to 30.53 kPa, allowing for stable adhesion to oral tissues for over 24 h. *In vitro* experiments demonstrated good cytocompatibility and promoted fibroblast adhesion and proliferation. *In vivo* studies using murine skin and rat oral mucosal defect models showed accelerated wound closure, reduced scar formation, and improved tissue regeneration. Immunological analyses revealed that the DA-modified nanofiber membrane modulated the local immune microenvironment by promoting macrophage polarization toward the anti-inflammatory M2 phenotype. Transcriptomic analysis and subsequent molecular validation identified activation of the peroxisome proliferator-activated receptor gamma (PPARγ) signaling pathway as a key mechanism underlying this immunoregulatory effect. These results demonstrate that the mussel-inspired coaxial nanofiber membrane provides stable wet adhesion and actively regulates immune responses to promote scarless healing of oral mucosal defects, highlighting its potential as a functional biomaterial for soft tissue repair.

## 1 Introduction

**Scheme 1:**
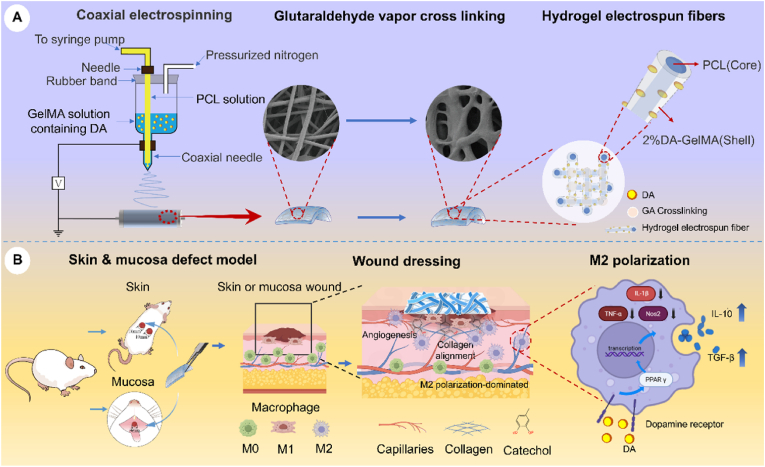
Design and application of the DA/PCL@GelMA nanofiber membrane. The DA/PCL@GelMA nanofiber membrane was fabricated via coaxial electrospinning using GelMA as the shell layer and PCL as the core layer, with dopamine incorporated into the shell to confer adhesive and immunomodulatory properties (A). The resulting membrane was applied to both dorsal skin defects and palatal mucosal defects in animal models, where it exhibited strong tissue adhesion and promoted tissue repair by regulating macrophage polarization (B).

Tumor resection, precancerous lesions, and trauma can all result in extensive oral mucosal defects. Among these causes, the prevalence of oral precancerous lesions in Asia was reported to be 10.54% [1], and in high-risk region of South Asia the prevalence can be as high as 14.84% [2], and the resulting mucosal defects significantly impair both oral function and facial appearance. Current clinical approaches for repairing oral mucosal defects include direct suturing, autologous skin grafting, flap transplantation, and the use of allogeneic decellularized dermal matrix tissue patches [3]. However, the oral cavity is characterized by a dynamically moist environment due to continuous physiological activities such as saliva secretion, mastication, swallowing, and speech. Under these conditions, most commercially available repair materials fail to maintain stable adhesion to the wound surface. Despite the use of bioactive materials to enhance healing, complications such as wound infection, incomplete epithelialization, and scar hyperplasia remain common [4], often leading to restricted mouth opening and reduced quality of life. Therefore, an ideal tissue-engineered membrane for oral mucosal repair should exhibit high mechanical toughness, support scar-free healing, possess excellent cell affinity and tissue adhesion [5], and maintain strong adhesive performance under dynamically moist conditions (see Scheme 1).

Inspired by biological adhesion mechanisms in nature, a range of biomimetic adhesive materials has been developed, including polydimethylsiloxane microcolumn arrays that mimic gecko setae [6], recombinant barnacle cement proteins [7], and snail mucus-inspired touch-responsive hydrogels [8]. However, these systems suffer from inherent limitations: gecko-inspired microstructures typically require low-humidity environments, barnacle cement proteins present challenges in extraction and degradation control, and snail mucus-inspired hydrogels often exhibit insufficient mechanical strength. In contrast, mussels can maintain robust and durable adhesion even in fully submerged and dynamic environments [9], making mussel-inspired strategies particularly suitable for the saliva-rich oral cavity. Mussel adhesion primarily depends on *Mytilus edulis* foot protein, in which dopamine (DA) is a key functional component responsible for strong wet adhesion [10]. DA-incorporated hydrogels have therefore been widely explored as tissue adhesives [11]. To simultaneously meet the mechanical and biocompatibility requirements of oral mucosal repair while providing an effective DA carrier, our group and others have demonstrated that coaxial electrospun fibers represent a promising strategy [12,13]. Electrospun nanofibers have attracted considerable attention due to their favorable mechanical properties and high porosity, which facilitates nutrient and metabolite exchange [14]. Among electrospinning substrates, synthetic polymers such as polycaprolactone (PCL) exhibit good biocompatibility, processability, and tunable mechanical properties, making them suitable for fibrous membranes [15]. However, PCL is limited by poor hydrophilicity and suboptimal cell affinity [16]. Gelatin methacrylate (GelMA), with its excellent hydrophilicity and biocompatibility, has therefore been used to modify PCL-based systems [17]. Compared with conventional blending or single-nozzle electrospinning, coaxial electrospinning enables complementary material integration, enhanced interfacial stability, and reduced shell detachment [14,18]. Core–shell fibrous scaffolds composed of PCL and GelMA have already demonstrated effectiveness in vascular applications [19], supporting the feasibility of this strategy for mucosal repair. Moreover, coaxial electrospun fibers provide an efficient platform for drug loading and controlled release [20], making them well suited for incorporating DA to further enhance tissue adhesion [21]. Accordingly, DA was introduced into the hydrogel shell layer in this study to improve adhesive performance.

It is well established that following biomaterial implantation, immune cells, rather than resident tissue cells, are the first to be recruited to the defect site [22], with macrophages representing the dominant early responders [23]. Macrophages play a central role in wound healing by transitioning from pro-inflammatory M1-like phenotypes to anti-inflammatory, pro-repair M2-like phenotypes, thereby regulating inflammation, fibrosis, and tissue regeneration [24]. As such, macrophage modulation has emerged as an effective therapeutic strategy to promote wound healing [25,26]; specifically, the immunomodulatory properties of DA have received increasing attention [27]. Existing studies indicate that DA can alleviate inflammation by activating DA receptors, leading to downregulation of nucleotide-binding oligomerization domain and leucine-rich repeat pyrin domain-containing protein 3 (NLRP3) expression and reduced production of inflammatory cytokines [28]. DA has also been shown to influence immune responses through the Janus kinase/signal transducer and activator of transcription (JAK/STAT) pathway, interleukin signaling, and peroxisome proliferator-activated receptor gamma (PPAR-γ) pathways [29], thereby upregulating genes associated with M2 macrophage polarization [30]. However, the specific mechanisms by which DA-modified biomaterials regulate macrophage behavior during oral mucosal wound healing remain insufficiently understood.

In this study, we developed a mussel-inspired adhesive nanofiber membrane using coaxial electrospinning, with PCL as the core and DA-modified GelMA as the shell. The physicochemical properties of the membrane, including tensile strength, swelling behavior, and degradation profile, were systematically characterized. Adhesive performance was evaluated through in vitro tissue adhesion assays and in vivo oral mucosal defect models. In addition, cytological experiments were conducted to assess biocompatibility, while animal studies were used to evaluate wound-healing efficacy and immunoregulatory effects. Transcriptomic sequencing combined with molecular biology analyses was employed to elucidate the underlying mechanisms. This work aims to provide a functional and mechanistically informed strategy for the repair of oral mucosal defects.

## 2 Material and methods

### 2.1 Design and fabrication of hydrogel nanofibers

Gelatin (1.0 g) was dissolved in phosphate-buffered saline (PBS, 10 mL) under magnetic stirring at 400 rpm in a 50 °C water bath. Methacrylic anhydride (0.8 mL) was then added dropwise, and the reaction was allowed to proceed for 3 h. The resulting solution was dialyzed against deionized water using dialysis tubing with a molecular weight cutoff of 4–8 kDa at 40 °C for 2 days. After dialysis, the gel solution was frozen overnight and subsequently freeze-dried at −80 °C to obtain GelMA. GelMA and PCL were separately dissolved in 2,2,2-trifluoroethanol (TFEA) to prepare spinning solutions at a concentration of 10% (w/v). During coaxial electrospinning, the shell-to-core flow rate ratios were set to 0.003 mm/s:0.001 mm/s (3:1), 0.0026 mm/s:0.0013 mm/s (2:1), and 0.002 mm/s:0.002 mm/s (1:1), respectively. The collected fibers were crosslinked in an ethanol solution containing 10% glutaraldehyde for 10 h at 28 °C, followed by thorough washing, swelling in deionized water, and freeze-drying to obtain the composite membrane. Unless otherwise stated, gelatin, methacrylic anhydrid, TFEA, and other reagents were obtained from Sigma-Aldrich (St. Louis, MO, USA).

### 2.2 Preparation of self-adhesive hydrogel nanofibers

DA was dissolved in 1 mL deionized water at amounts of 0.06 g, 0.12 g, and 0.18 g, respectively. Subsequently, 5 mL TFEA and 0.6 g GelMA were added, and the mixture was stirred magnetically to obtain GelMA spinning solutions (10% w/v) containing 1%, 2%, or 3% dopamine (w/v). The core spinning solution consisted of 10% (w/v) PCL dissolved in TFEA. After coaxial electrospinning, the nanofibrous membranes were crosslinked using glutaraldehyde vapor to stabilize the structure.

### 2.3 Morphology of hydrogel nanofibers

The surface morphology of the nanofiber membranes was examined using scanning electron microscopy (SEM) at an accelerating voltage of 3 kV. Prior to observation, samples were sputter-coated with gold at a constant current of 10 mA under vacuum for 120 s. Transmission electron microscopy (TEM) was employed to visualize the core-shell structure of the nanofibers prepared at different shell-to-core flow rate ratios.

### 2.4 Mechanical property test

The mechanical properties of the hydrogel nanofiber membranes were evaluated using a universal materials testing machine. Rectangular specimens with dimensions of 10 mm × 100 mm and a thickness of 50 μm were tested with a clamping distance of 30 mm at a tensile speed of 10 mm/min.

### 2.5 Swelling degree test

Rectangular specimens (10 mm × 100 mm) were cut from the crosslinked nanofiber membranes and immersed in deionized water for 24 h at room temperature. After immersion, excess surface water was gently removed using filter paper, and the wet weight (W_2_) was recorded. The samples were then freeze-dried to constant weight, and the dry weight (W_1_) was measured. The swelling degree (SD) was calculated using the following equation:

$$S_{d}=\frac{\left(W_{2}-W_{1}\right)}{W_{1}}\times 100\%$$

where W_1_ represents the weight of the freeze-dried sample and W_2_ represents the weight of the swollen sample after surface water removal.

### 2.6 Degradation test

Nanofiber membranes were cut into specimens of identical size, dried, and weighed to obtain the initial weight (Wᵢ). The samples were then immersed in PBS (pH 7.4) at room temperature. At predetermined time points (1, 4, 10, 17, 24, and 40 days), the membranes were removed from the solution, rinsed gently with deionized water, dried to constant weight, and weighed to obtain the remaining weight (W_t_). All experiments were performed in triplicate. The percentage of weight loss was calculated using the following equation:

$$Weight\;loss\;\left(\%\right)=\frac{\left(W_{2}-W_{1}\right)}{W_{1}}$$

where Wᵢ represents the initial dry weight of the nanofiber membrane and W_t_ represents the dry weight after immersion for the specified period.

### 2.7 Test of adhesion performance

Fresh porcine skin with a uniform thickness of 5 mm was used as the experimental substrate and kept moist with PBS. The porcine skin specimens were cut to dimensions of 2.5 cm × 5 cm, while the nanofiber membranes were cut to 1 cm × 2.5 cm. The membrane and substrate were overlapped in a straight-line configuration with a defined bonding area, as illustrated in Fig. 2A and B. After a standing period of 15 min to allow adhesion, mechanical testing was performed in accordance with YY/T 0729.1-2009, Test Method for Adhesive Bonding Performance of Tissue Adhesives—Part 1: Overlap Shear Tensile Strength. The maximum load required to gradually separate the bonded specimens was recorded, and the interfacial shear strength was calculated by dividing the maximum load by the bonding area. Adhesive strength was expressed in kilopascals (kPa).

### 2.8 Test of adhesion performance under wet conditions

For the short-term adhesion test, freshly excised rat organs, including heart, liver, kidney, and tongue, were rinsed with PBS and briefly air-dried. Slightly wetted self-adhesive hydrogel nanofiber membranes were cut to appropriate sizes, applied to the organ surfaces, and then immersed in artificial saliva. At predetermined time points, samples were removed from the solution, and the adhesion process was documented using a digital camera.

For the long-term adhesion test, rat liver tissue was used as the substrate. To preserve tissue integrity, liver samples covered with self-adhesive hydrogel nanofiber membranes were immersed in artificial saliva containing polylysine (400 mg/kg) as a preservative. Adhesion stability was recorded using a digital camera after immersion for 1, 3, 5, 7, 10, and 15 days.

### 2.9 Cell culture

Mouse fibroblast 3T3 cells were obtained from MeisenCTCC (Nanjing, Jiangsu, China). All cell culture procedures were conducted under sterile conditions. Cells were cultured in Dulbecco's modified Eagle's medium (DMEM; Gibco, Thermo Fisher Scientific, Waltham, MA, USA) supplemented with 10% fetal bovine serum and 1% penicillin–streptomycin (Gibco), and maintained at 37 °C in a humidified atmosphere containing 5% CO_2_.

### 2.10 Cytotoxicity of hydrogel nanofiber membrane leaching solution

To prepare material leaching solutions, nanofiber membranes (5 cm in length) of 2% DA–GelMA/PCL and 0% DA–GelMA/PCL were separately immersed in 10 mL complete culture medium and incubated at 37 °C for 24 h. Medium without material immersion served as the control. 3T3 cells were seeded into 96-well plates at a density of 1 × 10^5^ cells/mL (100 μL per well) and incubated at 37 °C with 5% CO_2_ until cell attachment. The culture medium was then replaced with the corresponding leaching solutions. After 6 h of incubation, Cell Counting Kit-8 reagent (Solarbio, Beijing, China) was added, and the absorbance at 450 nm was measured to assess cell viability (n = 5).

### 2.11 Cytotoxicity of hydrogel nanofiber membrane coculture system

Nanofiber membranes were fixed to the bottom of culture wells and prewetted with 1 mL PBS for 24 h 3T3 cells were adjusted to a density of 2 × 10^4^ cells/mL and seeded onto the membrane surface at 50 μL per well. After incubation at 37 °C with 5% CO_2_ for 30 min to allow cell attachment, 500 μL of complete culture medium was gently added along the wall of each well. Cells were cultured for 24, 48, 72, and 96 h. At each time point, 50 μL Cell Counting Kit-8 reagent (CCK-8; Solarbio) was added to each well and incubated for 1 h. Subsequently, 100 μL of supernatant from each well was transferred to a 96-well plate, and absorbance at 450 nm was measured to evaluate cell viability.

### 2.12 Cell viability assay

Leaching solution and membrane coculture systems were prepared with a seeding density of 5000 cells per well. After incubation for 24 and 48 h, the culture medium was removed and cells were rinsed three times with PBS. Cells were then incubated with 100 μL Calcein AM working solution (Beyotime Biotechnology, Shanghai, China) for 1 h at 37 °C. Live cells stained with green fluorescence were imaged using an inverted fluorescence microscope.

### 2.13 Cell morphology analysis

Cell morphology in both the leaching solution system and the nanofiber membrane coculture system was evaluated following the same preparation procedures described above. After 24 h of incubation, culture medium was discarded and cells were rinsed three times with PBS. Cells were fixed with 4% paraformaldehyde (Solarbio) for 10 min, permeabilized with 0.2% Triton X-100 (Solarbio) for 5 min, and then stained for F-actin using Actin Tracker Red Rhodamine (Beyotime Biotechnology) diluted 1:200 in antibody diluent. After incubation at room temperature in the dark for 30 min, nuclei were counterstained with DAPI (Beyotime Biotechnology) for 5–10 min. Cell morphology and cytoskeletal organization were observed using confocal laser scanning microscopy (Olympus, Tokyo, Japan).

### 2.14 Mouse dorsal skin wound repair assay in vivo

The ICR mice (SPF grade, 25-30g) were anesthetized with phenobarbital sodium(100 mg/kg) via intraperitoneal injections. Make 1 cm-diameter circular incisions on both sides of the dorsal skin of mice down to the subcutaneous tissue. The materials were adhered to the dorsal skin defects, and the wound conditions were photographed and recorded on days 0, 3, 7, 14 and 21, respectively. At different time points, the specimens were harvested and sent to Biosharp Biotechnology Co., Ltd. (China) for HE staining, Masson staining and immunohistochemical staining (CD31, SMA, Collagen Ⅰ and Collagen Ⅲ) and immunofluorescence staining (F4/80, CD86, CD206).

### 2.15 Rat palatal mucosa wound repair assay in vivo

The SD rats (SPF grade, 300-350g) were anesthetized with phenobarbital sodium(100 mg/kg) via intraperitoneal injections. Make a rectangular incision with a side length of approximately 4 mm on the mucosa of the rat. The materials were adhered to the palatal mucosa defects, and the wound conditions were photographed and recorded on days 1, 4, 7 respectively. At different time points, the specimens were harvested and sent to Biosharp Biotechnology Co., Ltd. (China) for HE staining, Masson staining.

### 2.16 Transcriptome analysis

On postoperative day 3, rats were euthanized, and palatal mucosal tissues were harvested from the adhesive membrane group (n = 3) and the blank control group (n = 3). The entire layer of newly formed mucosal tissue at the defect site, together with a small margin of adjacent normal tissue, was carefully excised. Tissue samples were immediately immersed in RNA extraction reagent and submitted for transcriptome sequencing to Shanghai Hongxu Biotechnology Co., Ltd. (Shanghai, China). Differential gene expression analysis was subsequently performed to identify genes with statistically significant changes between groups.

### 2.17 Culture of iBMDM

Immortalized bone marrow–derived macrophages (iBMDM; mice-M3-1001) were purchased from Oricell Biosciences Co., Ltd. (Guangzhou, China). Cells were rinsed with PBS and cultured in DMEM (Gibco), supplemented with 10% fetal bovine serum (FBS; Gibco) and antibiotics (1% penicillin–streptomycin; Solarbio). Cells were maintained at 37 °C in a humidified incubator with 5% CO_2_. For coculture experiments, 2% DA–GelMA/PCL and 0% DA–GelMA/PCL membranes were cut to appropriate sizes and placed flat at the bottom of 6-well plates. iBMDMs were seeded at a density of 1 × 10^5^ cells/mL, with 2 mL of cell suspension added to each well. After 24 h of incubation, GW9662 was added to the designated groups at a final concentration of 10 μM, while an equivalent volume of dimethyl sulfoxide was added to the vehicle control groups. Cells were then cultured for an additional 48 h.

### 2.18 Flow cytometry

iBMDMs from different treatment groups were collected and preincubated with TruStain FcX (Cat. No. 101319; BioLegend, San Diego, CA, USA) to reduce nonspecific Fc receptor binding. Cell viability and macrophage identity were assessed by staining with Fixable Viability Dye (Cat. No. 423106; BioLegend; 1:100), anti-F4/80–FITC (Cat. No. 123108; BioLegend; 1:100), and anti-CD11b–APC (Cat. No. 101212; BioLegend; 1:100). Cells were then stained with anti-CD86–PE-Cy7 (Cat. No. 105014; BioLegend; 1:100), followed by fixation and permeabilization, and subsequently incubated with anti-CD206–PE (Cat. No. 141706; BioLegend; 1:100). After washing, cells were resuspended and analyzed using a BD FACSCanto II flow cytometer (BD Biosciences, San Jose, CA, USA).

### 2.19 Quantitative polymerase chain reaction

Total RNA was extracted from iBMDMs in different treatment groups using a total RNA isolation kit (EZBioscience, Roseville, MN, USA), following the manufacturer's instructions. Complementary DNA was synthesized by reverse transcription, and quantitative polymerase chain reaction was performed using SYBR qPCR Master Mix (EZBioscience) on a CFX96 Real-Time PCR System (Bio-Rad, Hercules, CA, USA). Gene expression levels were normalized to *GAPDH* as the endogenous control. Primer sequences used for amplification are provided in Supplementary Table 1.

### 2.20 Western blot

Total protein was extracted from iBMDMs using a protein extraction kit (Solarbio). Protein samples were denatured for 10 min, separated by sodium dodecyl sulfate–polyacrylamide gel electrophoresis, and transferred onto polyvinylidene fluoride membranes (Millipore, Burlington, MA, USA). Membranes were blocked with 5% bovine serum albumin and incubated overnight at 4 °C with primary antibodies listed in Supplementary Table 2. The membranes were then incubated with horseradish peroxidase–conjugated secondary antibodies for 1 h at room temperature. Protein bands were visualized using the ChemiDoc XRS + imaging system (Bio-Rad) and quantified with ImageJ software (National Institutes of Health, Bethesda, MD, USA), with signal intensities normalized to the corresponding controls. Primary antibodies against IL1β (Cat. No. YP-16004), IL10 (Cat. No. YP-15986), PPARγ (Cat. No. mAb-03325), and GAPDH (Cat. No. mAb-03525) were purchased from YOPIN Bio (Shanghai, China).

### 2.21 Statistical analysis

Quantitative data are presented as mean ± standard deviation. Statistical analyses were performed using SPSS version 26.0 (IBM Corp., Armonk, NY, USA) and GraphPad Prism version 9.0 (GraphPad Corp., San Diego, CA, USA). Comparisons between two groups were conducted using the Student's *t*-test, while one-way analysis of variance followed by Tukey's post hoc test was used for comparisons among multiple groups. A p value < 0.05 was considered statistically significant. Statistical significance is indicated as ∗*P* < 0.05, ∗∗*P* < 0.01, and ∗∗∗*P* < 0.0001.

## 3 Results and discussion

### 3.1 Adhesion performance tests of self-adhesive hydrogel membranes

**Fig. 1:**
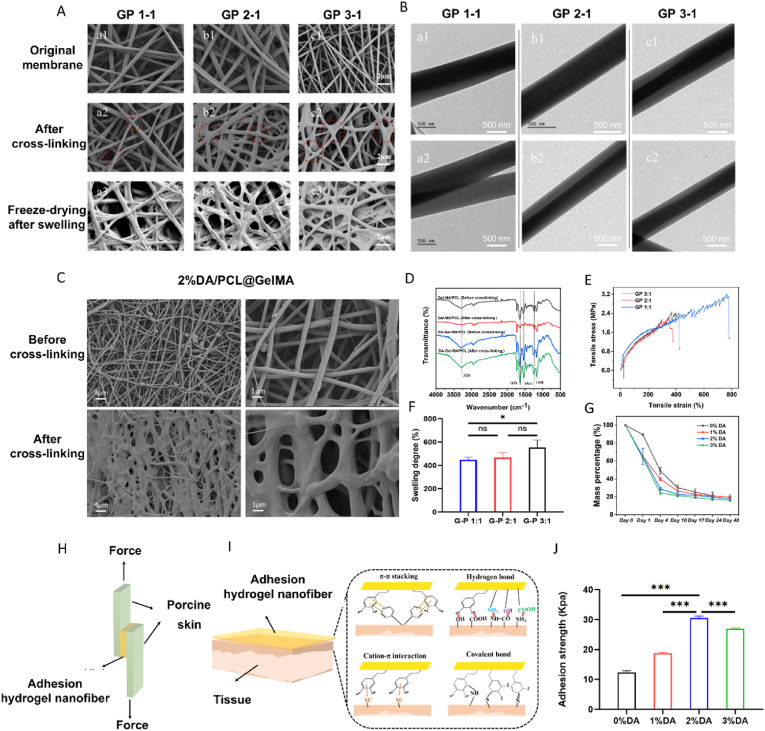
Morphological characterization and adhesive performance of the nanofiber membrane. Scanning electron microscopy (SEM) images show the morphology of coaxial nanofibers prepared at different shell-to-core flow rate ratios (A), while transmission electron microscopy confirms the corresponding core–shell structures (B). SEM images demonstrate that dopamine incorporation does not disrupt nanofiber morphology (C). Fourier transform infrared spectra illustrate the characteristic chemical bonds of hydrogel nanofibers with different dopamine contents (D). Stress–strain curves reveal the mechanical behavior of hydrogel nanofibers with varying dopamine concentrations (E). The swelling degree of nanofibers prepared at different shell–core flow ratios is shown, analyzed by one-way ANOVA (n = 3) (F). Degradation profiles of hydrogel fiber membranes with different dopamine contents in PBS (pH 7.4) are presented, analyzed by one-way ANOVA (n = 3) (G). A schematic illustration of the porcine skin overlap shear test is shown (H), along with the proposed adhesion mechanism between the hydrogel nanofiber membrane and tissue (I). Quantitative adhesion strengths of hydrogel fiber membranes with different dopamine contents are summarized and analyzed by one-way ANOVA (n = 3) (J).

Coaxial electrospinning was employed using GelMA as the shell layer and PCL as the core layer. When the shell-to-core flow rate ratio was set at 3:1 (GP 3–1), the resulting nanofibers exhibited the most uniform morphology and well-defined core–shell structure, as confirmed by TEM (Fig. 1A), with complete encapsulation of the core by the shell layer (Fig. 1B). In contrast, non-coaxial architectures, including parallel or offset fibers, were frequently observed at other flow rate ratios. SEM analysis indicated that DA incorporation did not adversely affect the formation of coaxial nanofibers (Fig. 1C). FT-IR spectra revealed characteristic peaks corresponding to C=O, N–H, and C–N bonds, indicating that the crosslinking process had minimal impact on the electrospun fiber structure (Fig. 1D). With increasing GelMA content, tensile strength gradually decreased, whereas swelling capacity increased. The GP 3–1 nanofiber membrane exhibited a swelling degree of 554.6% (Fig. 1E), an elongation at break of 464.33%, and a tensile strength of 2.52 MPa (Fig. 1F), while also demonstrating favorable degradability (Fig. 1G).

**Fig. 2:**
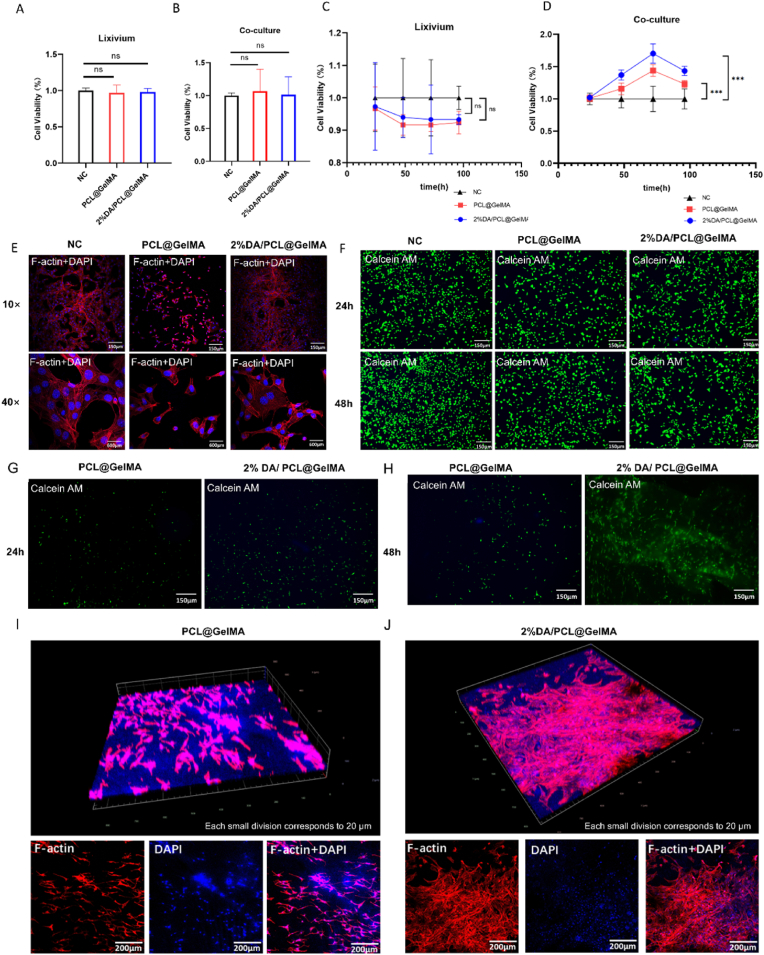
Cytological evaluation of the biocompatibility of nanofibrous membranes. Cell viability of 3T3 fibroblasts treated with membrane leachates (A) or cocultured with hydrogel nanofiber membranes (B) for 6 h is shown, analyzed by one-way ANOVA (n = 3). Proliferation rates of 3T3 cells treated with leachates (C) or cocultured with membranes (D) over 1–4 days are presented, analyzed by two-way ANOVA (n = 3). Live-cell staining illustrates cell viability after treatment with leachates (E). F-actin immunofluorescence staining demonstrates cytoskeletal organization and morphological changes induced by membrane leachates (F). Live-cell staining of 3T3 cells cocultured with nanofiber membranes for 24 h (G) and 48 h (H) is shown. Three-dimensional distribution and invasion of 3T3 cells within the PCL@GelMA membrane (I) and the 2% DA/PCL@GelMA membrane (J) after 48 h are illustrated.

The adhesive strength of hydrogel nanofiber membranes containing 0%, 1%, 2%, and 3% DA toward porcine skin tissue was 12.36, 18.76, 30.53, and 26.92 kPa, respectively (Fig. 1H–J). The membrane containing 2% DA exhibited the highest adhesive strength. The declined adhesion performance of the 3% DA sample may be related to its reduced porosity after crosslinking, and its correlation with molecular-level structural changes remains to be further explored. Furthermore, nanofiber membranes adhered to rat heart, liver, kidney, and tongue tissues remained firmly attached after immersion in artificial saliva containing polylysine (400 mg/kg) for up to 15 days (Fig. 2F), demonstrating stable adhesion under humid physiological conditions. Although the material swells significantly, it exhibits uniform thickness-dominant swelling with negligible change in planar area, showing no obvious warping, curling, or excessive dimensional deformation.

The introduction of DA significantly enhanced the bonding strength of the GelMA/PCL nanofiber membrane. As the FTIR showed, the oxidative polymerization of DA proceeds continuously. Therefore, it can also be referred to as a DA-related interface-modified material. As a small molecule rich in catechol groups, DA can adhere to diverse substrates through covalent and noncovalent interactions. Notably, the adhesive strength of the 2% DA-modified membrane reached 30.53 kPa, substantially exceeding that of commercially available fibrin glue (≈4 kPa) [31]. Compared with recently reported oral mucosal adhesive materials, the adhesive performance achieved in this study is within an optimal range (Table 1). Additionally, underwater biosensor materials achieved ultra-fast swelling and instant adhesion through polymer interpenetrating porous network structures, are promising research directions as well [36].

**Table 1:** Comparison of adhesive properties of oral mucosal adhesive materials reported in the past two years.

Author	Year of publication	Biomaterial	Wet adhesive strength
Liu et al. [32]	2023	GelMA-methacrylate hyaluronic acid hydrogel + tannic acid + polydopamine modified zinc oxide	46.25 ± 3.425 kPa
An et al. [33]	2022	Janus gelatin-polydopamine-nanoclay hydrogel	63 kPa
Zhu et al. [16]	2022	GelMA-nanoclay-tannic acid hydrogel	>15 kPa
Yi et al. [34]	2024	Polydimethylacrylamide/Carboxymethyl Chitosan Hydrogel	18.7 kPa
Li et al. [35]	2024	Cationicmonomer [2-(acryloyloxy) ethyl] + trimethylammonium chloride	18.67 kPa

Zhang et al. [37] developed a light-responsive hydrogel based on cyclic nitrobenzene–modified hyaluronic acid, which achieved a bonding strength of 16 kPa and maintained adhesion for up to 24 h under wet conditions. In the present study, the adhesion performance of the 2% DA/PCL@GelMA hydrogel nanofiber membrane was evaluated in a rat model that more closely mimics the complex oral environment, and stable adhesion was similarly maintained for more than 24 h. In addition to adhesion, appropriate degradability is critical for oral mucosal repair materials. By day 4, the mass of the 2% DA/PCL@GelMA membrane had decreased to 28.85% of its initial value. Previous studies have shown that significant epithelial cell migration occurs approximately 4 days after injury [38], indicating that the degradation profile of the membrane is well matched to the mucosal healing process. After hydrogel degradation, the residual PCL scaffold provides sustained structural support and facilitates cell infiltration and nutrient exchange. More researches are required to accelerate PCL degradation to prevent foreign body reactions and inflammation. These findings demonstrate that the 2% DA/PCL@GelMA hydrogel nanofiber membrane combines favorable mechanical properties with particularly strong and durable adhesion, supporting its potential application in oral mucosal defect repair.

### 3.2 *In vitro* biocompatibility of self-adhesive hydrogel membranes

Cytotoxicity assessed by the CCK-8 assay (Fig. 2A and B) showed that 3T3 cell viability was not significantly reduced after treatment with membrane leaching solutions or coculture with hydrogel nanofiber membranes for 6 h. Consistently, cell proliferation assays (Fig. 2C and D) revealed no significant difference between the control group and cells treated with leaching solutions (P > 0.05). In contrast, in the coculture system, cells cultured on the 2% DA/PCL@GelMA membrane exhibited significantly enhanced proliferative activity compared with the control group on day 3 (P < 0.05), which can be attributed not only to the increased surface area provided by the nanofibrous structure but also to its favorable biocompatibility.

Cell viability and morphology were further evaluated using Calcein AM staining. As shown in Fig. 2E, cells cultured with leachates from both 2% DA/PCL@GelMA and PCL@GelMA membranes displayed normal morphology at 24 and 48 h. Although the density of viable cells in the leachate-treated groups was slightly lower than that in the control group, this difference was consistent with the nonsignificant CCK-8 results. In the coculture system, 3T3 cells exhibited a clear proliferative trend from 24 to 48 h (Fig. 2G and H), with a higher viable cell density observed on the 2% DA/PCL@GelMA membrane compared with the PCL@GelMA membrane. Confocal microscopy revealed that cells exposed to PCL@GelMA leachates showed mildly irregular morphology and disorganized cytoskeletal structures, whereas cells in the 2% DA/PCL@GelMA and normal control groups maintained well-organized actin cytoskeletons and normal morphology (Fig. 2F). Tomographic imaging of the coculture system further demonstrated that most cells migrated to a depth of approximately 100 μm within the hydrogel nanofiber membrane after 48 h (Fig. 2I and J), with significantly greater cell proliferation observed in the 2% DA/PCL@GelMA group than in the PCL@GelMA group. These findings indicate that DA modification facilitates cell migration and colonization within the nanofibrous scaffold.

### 3.3 *In vivo* biocompatibility of self-adhesive hydrogel membranes

In the animal study, two full-thickness skin defects (1 cm in diameter) were created on the dorsal region of each mouse. A total of 48 wounds were randomly divided into four groups (n = 12 per group): wounds covered with 2% DA/PCL@GelMA hydrogel nanofiber membranes (experimental group), wounds treated with a commercial adhesive tape (Bonanga, Beijing, China) as the positive control, wounds covered with GelMA/PCL nanofiber membranes as the negative control, and wounds left untreated as the blank control. Bonanga consists of hydrophilic cellulose and hydrophobic medical polymer components and can adhere stably to intraoral wounds for more than 24 h while preventing contamination.

**Fig. 3:**
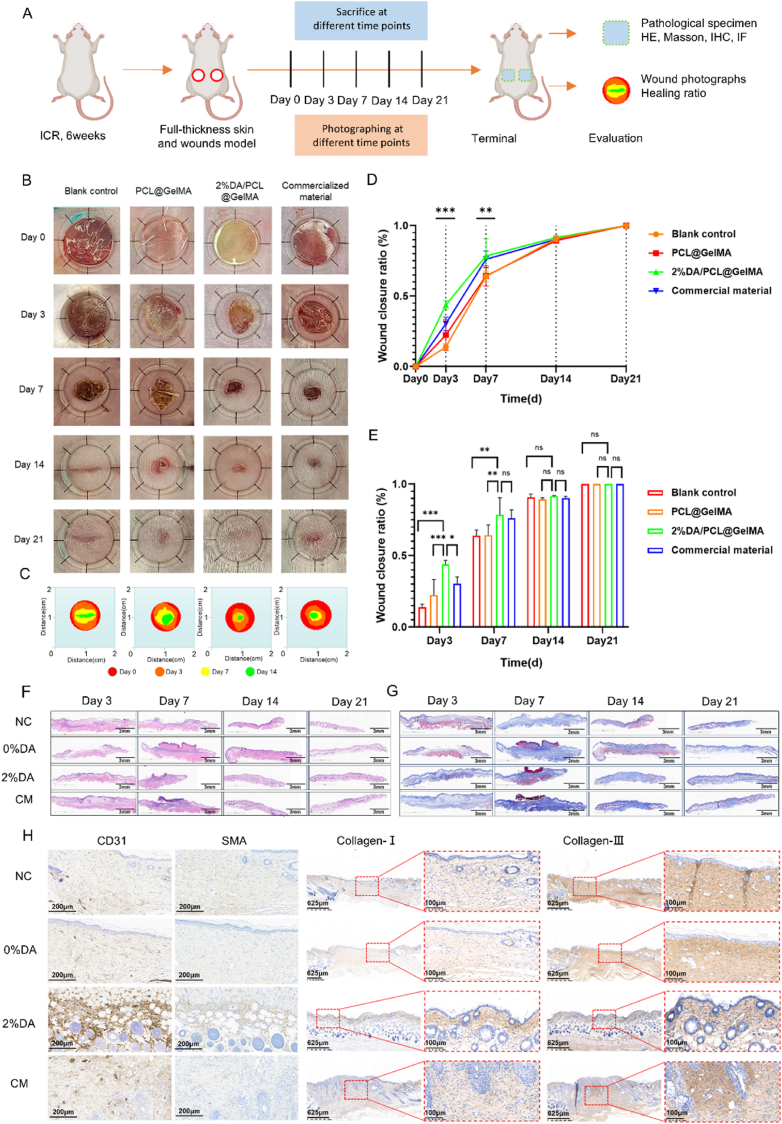
Wound healing performance of nanofibrous membranes in a mouse dorsal skin defect model. A schematic diagram and experimental timeline of the full-thickness dorsal skin defect model are shown (A). Representative images depict the wound healing process in each treatment group over time (B). Quantitative analysis of wound healing trajectories is presented (C), along with temporal changes in wound closure rates (D) and comparative wound healing rates among groups at different time points, where ∗*P* < 0.05, ∗∗*P* < 0.01, and ∗∗∗*P* < 0.001, analyzed by one-way ANOVA (n = 3) (E). Hematoxylin and eosin staining (F) and Masson's trichrome staining (G) illustrate histological features of wound tissues harvested at postoperative days 3, 7, 14, and 21. Immunohistochemical staining of α-SMA, CD31, collagen I, and collagen III demonstrates vascularization and extracellular matrix remodeling at day 21 after surgery (H).

Wound healing was monitored by digital photography at 3, 7, 14, and 21 days postoperatively (Fig. 3A). Wound areas were quantified using ImageJ software, and wound closure rates were calculated (Fig. 3B–E). All groups exhibited progressive wound contraction over time (Fig. 3B). Notably, the experimental group demonstrated the most rapid wound closure and the least scar formation, with significantly improved healing observed on days 3 and 7, indicating that the 2% DA/PCL@GelMA membrane effectively promoted early wound closure. By day 21, wounds in all groups were nearly completely healed; however, the experimental group exhibited a smooth, intact epithelium with minimal scarring, whereas the blank control group displayed evident linear scars. Overall, the healing outcome ranked highest in the experimental group, followed by the positive and negative control groups.

Full-thickness regenerated tissues along with a 5 mm margin of surrounding normal tissue were harvested for hematoxylin and eosin staining, Masson's trichrome staining, and immunohistochemical analysis of CD31, α-SMA, collagen I, and collagen III. Hematoxylin and eosin staining revealed continuous epithelial layers in all groups by day 14, with the appearance of newly formed skin appendages, including hair follicles, in the experimental group (Fig. 3F), a hallmark of scar-free healing. By day 21, the experimental group exhibited intact epithelial architecture and dense, uniformly distributed skin appendages, closely resembling normal skin, whereas the other groups showed delayed tissue regeneration. Masson's trichrome staining further demonstrated that collagen fibers in the experimental group were arranged in a parallel, uniform, and continuous manner, similar to normal tissue (Fig. 3G). In contrast, collagen fibers in the positive control group displayed a vortex-like pattern, while the negative and blank control groups exhibited sparse and disorganized collagen deposition.

Immunohistochemical staining for α-SMA and CD31, markers of vascular smooth muscle cells and endothelial cells, respectively, revealed increased neovascularization across all groups by day 21, with more mature and well-organized vascular structures observed in the experimental group (Fig. 3H). This finding suggests that the 2% DA/PCL@GelMA membrane promotes angiogenesis and vascular maturation, facilitating healthy granulation tissue formation. Consistently, collagen I and collagen III staining showed orderly and evenly distributed collagen fiber bundles in the experimental group, comparable to adjacent normal tissue, whereas collagen deposition in the negative and blank control groups remained sparse and disorganized. These histological findings align with the macroscopic observation of scar-free healing in the adhesive membrane–treated wounds.

### 3.4 Self-adhesive hydrogel membranes in mucosal wound healing and immunofluorescence

**Fig. 4:**
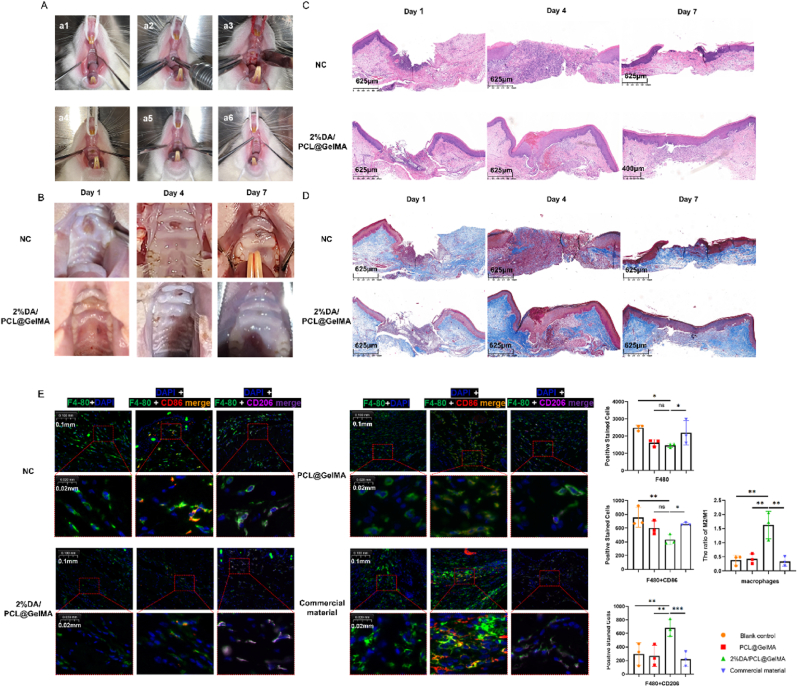
Repair of palatal mucosal defects by nanofibrous membranes in rats. The surgical procedure and adhesion performance of the rat palatal mucosal defect model are illustrated, with the final three images showing 6, 12, and 24 h after defect coverage with the adhesive membrane (A). Representative images show mucosal wound healing in different groups at postoperative days 1, 4, and 7 (B). Hematoxylin and eosin staining (C) and Masson's trichrome staining (D) reveal histological changes in mucosal tissues at corresponding time points. Immunofluorescence staining of F4/80, CD86, and CD206 demonstrates macrophage infiltration and polarization at the wound site 7 days after surgery, with quantitative analysis of positive stained cells and M2/M1 ratio performed using one-way ANOVA (n = 3) (E).

Twenty-four Sprague–Dawley rats were randomly assigned to an experimental group or a blank control group. A circular full-thickness mucosal defect (3 mm in diameter) was created at the center of the palatal mucosa (Fig. 4A). Wounds in the experimental group were covered with the 2% DA/PCL@GelMA membrane, whereas wounds in the blank group received no treatment. Wound healing was documented by photography at 1, 4, and 7 days postoperatively. As shown in Fig. 4B, the 2% DA/PCL@GelMA membrane adhered tightly to the wound surface and remained stably attached for more than 24 h. By day 7, wounds in the experimental group were largely closed, and the regenerated mucosal epithelium closely resembled the surrounding normal tissue. In contrast, wounds in the blank group had not fully healed and were covered with a gray pseudomembrane. At each time point, three rats per group (n = 3) were euthanized, and full-thickness healing tissues together with adjacent normal tissue were harvested for histological analysis. Hematoxylin and eosin staining showed more rapid epithelial migration from the wound margins and faster wound closure in the membrane-treated group. By day 7, wounds in the experimental group had healed completely without obvious scarring, whereas wounds in the blank group remained incompletely epithelialized (Fig. 4D). Masson's trichrome staining further revealed regularly aligned collagen fiber bundles in the experimental group, while collagen fibers in the blank group appeared relatively coarse and disorganized (Fig. 4C). These results indicate that the 2% DA/PCL@GelMA membrane effectively promotes oral mucosal wound healing and reduces scar hyperplasia.

Wound healing is a dynamic and multistage process that includes hemostasis, inflammation, proliferation, and remodeling [39]. Angiogenesis, defined as the formation of new vascular branches from existing vessels, plays a critical role in restoring microcirculation and tissue perfusion during healing [40], [41]. In the early healing phase, a greater number of neovascularized vessels with larger diameters and clearer structures were observed in the experimental group compared with the control group, contributing to the formation of healthier granulation tissue and facilitating tissue repair.

Immunofluorescence staining for F4/80, CD86, and CD206 was performed on skin wound tissues at 7 days postoperatively (Fig. 4E). In the experimental group treated with the 2% DA/PCL@GelMA membrane, macrophage accumulation at the wound site was markedly reduced compared with the other groups. Moreover, the experimental group exhibited fewer CD86-positive cells and a higher proportion of CD206-positive cells, indicating a shift toward M2 macrophage polarization. In contrast, this immunomodulatory effect was not observed in wounds treated with DA-free membranes, suggesting that DA is the primary contributor to macrophage regulation. Immune responses play a pivotal role in wound healing, as monocytes migrate to the injury site and differentiate into macrophages within 48–72 h, adopting distinct functional phenotypes at different healing stages [42,43]. Macrophages typically transition from a pro-inflammatory M1 phenotype to a pro-repair M2 phenotype during tissue regeneration [44]. In the skin defect model, macrophages in the experimental group predominantly exhibited an M2 phenotype by day 7, with a significantly higher M2 ratio than in the other groups. Excessive or prolonged inflammatory responses can impair tissue regeneration, delay re-epithelialization, and ultimately lead to hypertrophic scarring and fibrosis [[45], [46], [47]]. Consistent with these findings, Ballestas et al. [48] reported that a nanofibrous scaffold loaded with the immunomodulatory agent FTY720 increased M2 macrophage polarization and promoted hard palate mucosal healing. Cause the early inflammatory stage (day 3) was not involved, the dynamic transition process of macrophage phenotypes therefore remained to be further investigated. Collectively, these results suggest that the 2% DA/PCL@GelMA hydrogel nanofibrous membrane facilitates macrophage polarization toward the M2 phenotype, accelerates the transition from inflammation to tissue repair, and thereby enhances mucosal wound healing.

### 3.5 Transcriptome sequencing reveals potential mechanisms underlying enhanced healing

**Fig. 5:**
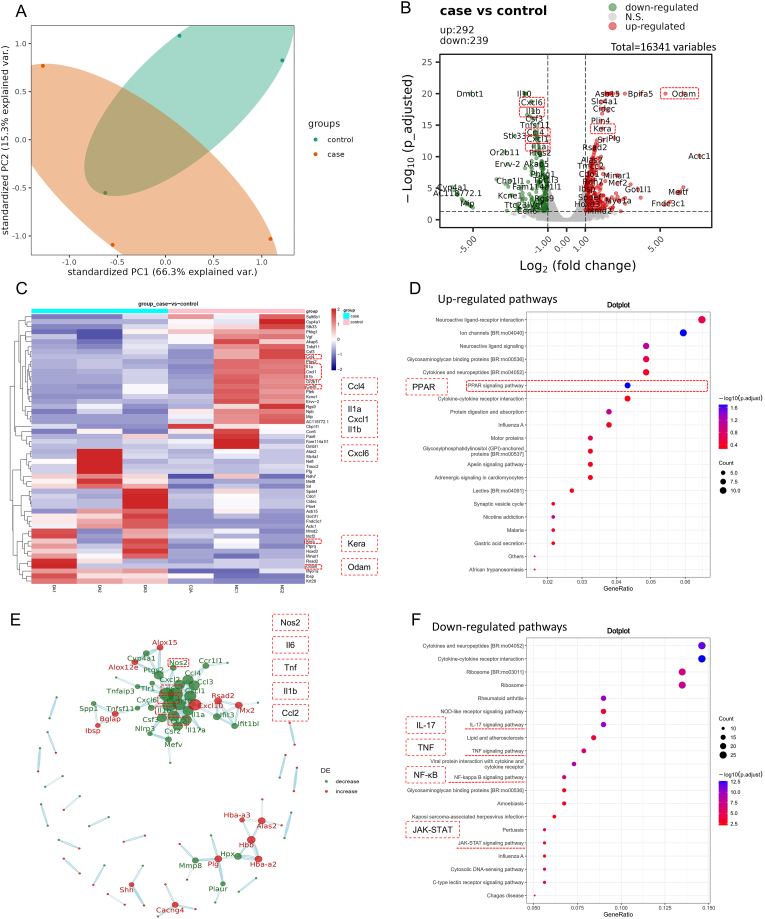
Transcriptomic analysis of palatal mucosal repair tissue. Principal component analysis demonstrates clear separation and good reproducibility between the adhesive membrane group and the blank group (A). A volcano plot illustrates the distribution of upregulated and downregulated differentially expressed genes between groups (B). A heatmap shows the expression profiles of 450 differentially expressed genes comparing the 2% DA/PCL@GelMA group with the blank group (C). KEGG pathway enrichment analysis identifies pathways upregulated (D) and downregulated (F) in the experimental group. A protein–protein interaction network of the top 300 genes highlights key regulatory clusters involved in immune and inflammatory responses (E).

Palatal mucosal defect tissues together with adjacent normal tissues were subjected to transcriptome sequencing to investigate the molecular mechanisms underlying the pro-healing effects of the 2% DA/PCL@GelMA membrane. Principal component analysis demonstrated good intragroup reproducibility and clear separation between the control group (NC) and the 2% DA/PCL@GelMA group, indicating distinct gene expression profiles between the two groups (Fig. 5A). A total of 531 differentially expressed genes were identified, including 292 upregulated and 239 downregulated genes in the experimental group compared with the blank group (Fig. 5B and C). Volcano plot analysis revealed significant upregulation of genes associated with keratin regeneration, such as *Kera* [49] and *Odam* [50], whereas multiple immune-related genes, including *IL1b*, *IL1a*, *Cxcl1*, *Cxcl6*, and *Ccl4*, were markedly downregulated [[51], [52], [53]]. These expression patterns highlight the pronounced immunomodulatory effects of the 2% DA/PCL@GelMA membrane and suggest macrophages as key target cells.

KEGG pathway enrichment analysis further supported these findings. Several inflammation-associated pathways, including rheumatoid arthritis, IL-17 signaling, TNF signaling, JAK–STAT, and NF-κB pathways, were significantly downregulated in the experimental group (Fig. 5D and F), indicating effective suppression of inflammatory responses. In contrast, the peroxisome proliferator-activated receptor (PPAR) signaling pathway was significantly upregulated (Fig. 5F), involving eight differentially expressed genes and exhibiting the lowest adjusted p value (p.adjust = 0.019) among all enriched pathways. And the genes localized to this pathway in KEGG analysis are predominantly recognized as downstream target genes of PPARγ. The PPARγ pathway is a well-established anti-inflammatory signaling axis and can inhibit inflammatory gene expression through interactions with transcription factors such as NF-κB [54] and activator protein-1 [32]. The prominent enrichment of this pathway suggests that PPAR signaling may play a central role in mediating the immunomodulatory effects of the 2% DA/PCL@GelMA membrane.

Gene co-expression network analysis revealed that the upregulated genes were mainly associated with metabolic processes [33] and neural signaling pathways [55] (Fig. 5E). The densest interaction network comprised genes such as *IL6*, *Tnf*, and *Ccl2*, indicating that immune and inflammatory regulation represents a core biological process in the experimental group. Several pro-inflammatory markers associated with macrophage M1 polarization and inflammatory chemotaxis, including *IL6*, *IL1b*, *NOS2*, *Tnf*, and members of the *Cxcl* family, were concurrently downregulated [56]. In addition, Ccl2, a key chemokine involved in macrophage recruitment [57], also showed reduced expression. The coordinated suppression of these genes suggests that the 2% DA/PCL@GelMA membrane inhibits M1 macrophage polarization and inflammatory cell recruitment, thereby creating a microenvironment conducive to tissue repair.

### 3.6 Cytological experiments on the immunomodulatory properties of the materials

**Fig. 6:**
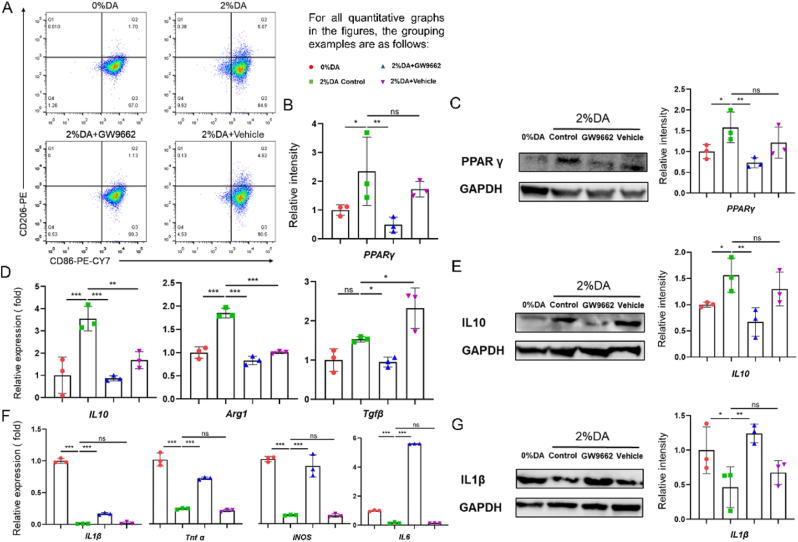
DA-induced activation of the PPARγ pathway promotes macrophage M2 polarization. Flow cytometric analysis shows the proportions of M1 and M2 macrophages in the 0% DA and 2% DA groups treated with vehicle or the PPARγ inhibitor GW9662 (A). qPCR (B) and Western blot analyses with quantification (C) demonstrate PPARγ expression levels (n = 3). Expression of M2-associated markers is shown by qPCR (D) and Western blot analysis with quantification (E) (n = 3). Expression of M1-associated markers is presented by qPCR (F) and Western blot analysis with quantification (G) (n = 3). Data are expressed as mean ± SD; ns indicates no significance; ∗*P* < 0.05, ∗∗*P* < 0.01, ∗∗∗*P* < 0.001.

Flow cytometry analysis demonstrated that coculture with different materials significantly affected iBMDM polarization (Fig. 6A). Compared with the 0% DA/PCL@GelMA group, macrophages cocultured with the 2% DA/PCL@GelMA membrane exhibited a markedly increased proportion of M2 polarization and a concomitant reduction in M1 polarization. Notably, treatment of the 2% DA/PCL@GelMA coculture group with GW9662, a specific PPARγ inhibitor, reversed the M2 polarization trend. GW9662 was dissolved in DMSO, and treatment with DMSO alone did not affect macrophage polarization. Consistent with the flow cytometry results, qPCR analysis showed significantly elevated PPARγ mRNA expression in both the 2% DA group and the 2% DA + Vehicle group (Fig. 6B), a finding further confirmed by Western blot analysis (Fig. 6C).

The expression of macrophage polarization markers was subsequently examined. M2-associated markers, including *IL10*, Arg*1*, and *Tgf-β*, were significantly upregulated in the 2% DA coculture group (Fig. 6D), whereas this upregulation was markedly attenuated following GW9662 treatment. Western blot analysis further demonstrated increased IL-10 protein expression in the 2% DA/PCL@GelMA coculture group (Fig. 6E). In contrast, the expression of M1-associated markers, including *IL1β*, *IL16*, *Tnf-α*, and *Nos2*, was significantly reduced in the 2% DA/PCL@GelMA group (Fig. 6F), and this suppression was reversed upon inhibition of PPARγ, as confirmed by both qPCR and Western blot analyses (Fig. 6G).

These cytological experiments demonstrate that 2% DA modification effectively induces macrophage polarization toward the M2 phenotype while suppressing M1 polarization, and that this immunomodulatory effect is dependent on activation of the PPARγ signaling pathway. Macrophages were initially identified as key target cells through immunofluorescence staining and transcriptomic analyses, and candidate signaling pathways were subsequently screened using KEGG enrichment analysis. The present findings validate these predictions at the cellular level and indicate that DA is a critical functional component responsible for the immunomodulatory activity of the DA/PCL@GelMA membrane. Previous studies have reported that DA can promote macrophage M2 polarization via dopamine receptors [58] and DA can modulate cellular physiological processes mainly by activating the DA1 receptor-linked PLC pathway, which elevates intracellular calcium levels and thereby upregulates the expression of PPAR family proteins [59]. Moreover, PPARγ is a well-established anti-inflammatory regulator [60] that can drive M2 polarization [61] through downstream pathways such as NFκB [60], STAT6 [62], and Akt/mTOR [63]. Building on this evidence, the present study confirms that DA modification activates PPARγ signaling to regulate macrophage polarization, thereby contributing to reduced inflammation and enhanced tissue repair. It remains unclear whether the observed effects are mediated by DA itself or by DA-derived oxidized or polymerized substances at the material interface, along with their associated signaling pathways with PPARγ. This represents a notable limitation of the present study. Future studies will focus on elucidating the specific DA–PPARγ interaction mechanisms to further guide the development of immunomodulatory biomaterials for inflammation control and scar-free tissue regeneration.

## 4 Conclusions

In summary, this study successfully developed a mussel-inspired, highly efficient self-adhesive hydrogel nanofiber membrane with a core-shell architecture via coaxial electrospinning, incorporating DA as a functional component. This biomaterial exhibits several notable advantages. First, it demonstrates favorable mechanical strength and robust adhesive performance under wet conditions, along with an appropriate swelling ratio and controlled degradability, enabling long-term structural stability and close conformity to wounds in the complex oral environment. Second, the membrane shows excellent biocompatibility and cell affinity, supporting cell adhesion, migration, and proliferation. Third, it maintains stable adhesion to oral mucosal wounds despite dynamic mechanical stresses such as traction, expansion, and torsion of surrounding tissues, and effectively promotes scar-free healing in both skin and oral mucosal defect models. Finally, the material exerts pronounced immunomodulatory effects by inhibiting excessive inflammation and promoting macrophage polarization toward the M2 phenotype, a process mediated by DA-induced activation of the PPARγ signaling pathway. Although the detailed upstream and downstream regulatory mechanisms warrant further investigation, this work provides a promising design strategy for adhesive wound dressings capable of promoting scar-free oral mucosal repair and improving functional outcomes in clinical applications.

## CRediT authorship contribution statement

**Junpeng Chen:** Conceptualization, Data curation, Formal analysis, Investigation, Methodology, Project administration, Resources, Software, Supervision, Validation, Visualization, Writing – original draft, Writing – review & editing. **Jinpeng Jiang:** Data curation, Investigation, Methodology, Project administration. **Wenyi Shen:** Data curation, Investigation, Methodology. **Bingjie Xu:** Conceptualization, Project administration, Resources, Software, Supervision, Validation. **Haina Miao:** Data curation, Investigation, Methodology. **Yangxi Cheng:** Data curation, Investigation, Methodology. **Yi Zheng:** Data curation, Investigation, Methodology. **Huiyong Zhu:** Project administration, Resources, Supervision. **Dan Yu:** Conceptualization, Data curation, Formal analysis, Funding acquisition, Investigation, Methodology, Project administration, Resources, Software, Supervision, Validation, Visualization, Writing – original draft, Writing – review & editing.

## Declaration of competing interest

The authors declare that they have no known competing financial interests or personal relationships that could have appeared to influence the work reported in this paper.

## Figures and Tables

**Appendix Fig. 1:**
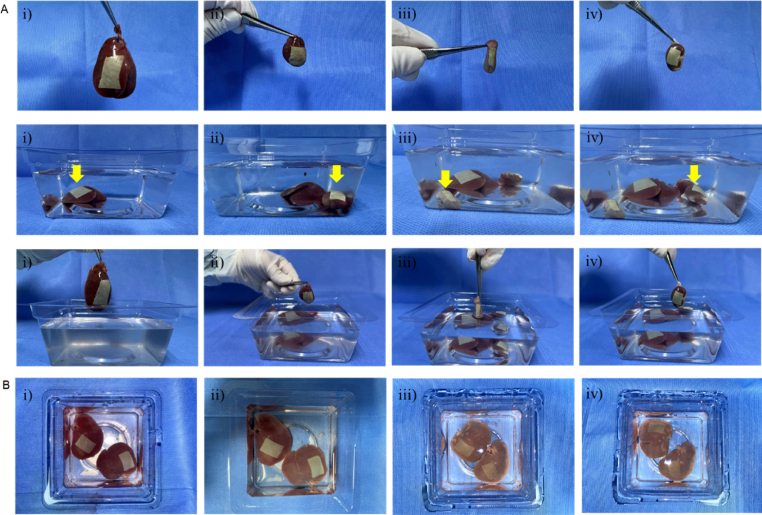
Adhesive performance of 2% DA/PCL@GelMA on organ surfaces. The adhesion behavior of the 2% DA/PCL@GelMA membrane on rat organ surfaces, including liver (i), heart (ii), tongue (iii), and kidney (iv), is shown, with images illustrating membrane attachment both in artificial saliva and after removal from the liquid environment (A). Long-term adhesion performance on rat liver tissue at 1, 3, 5, and 7 days is demonstrated (B).

**Appendix Fig. 2:**
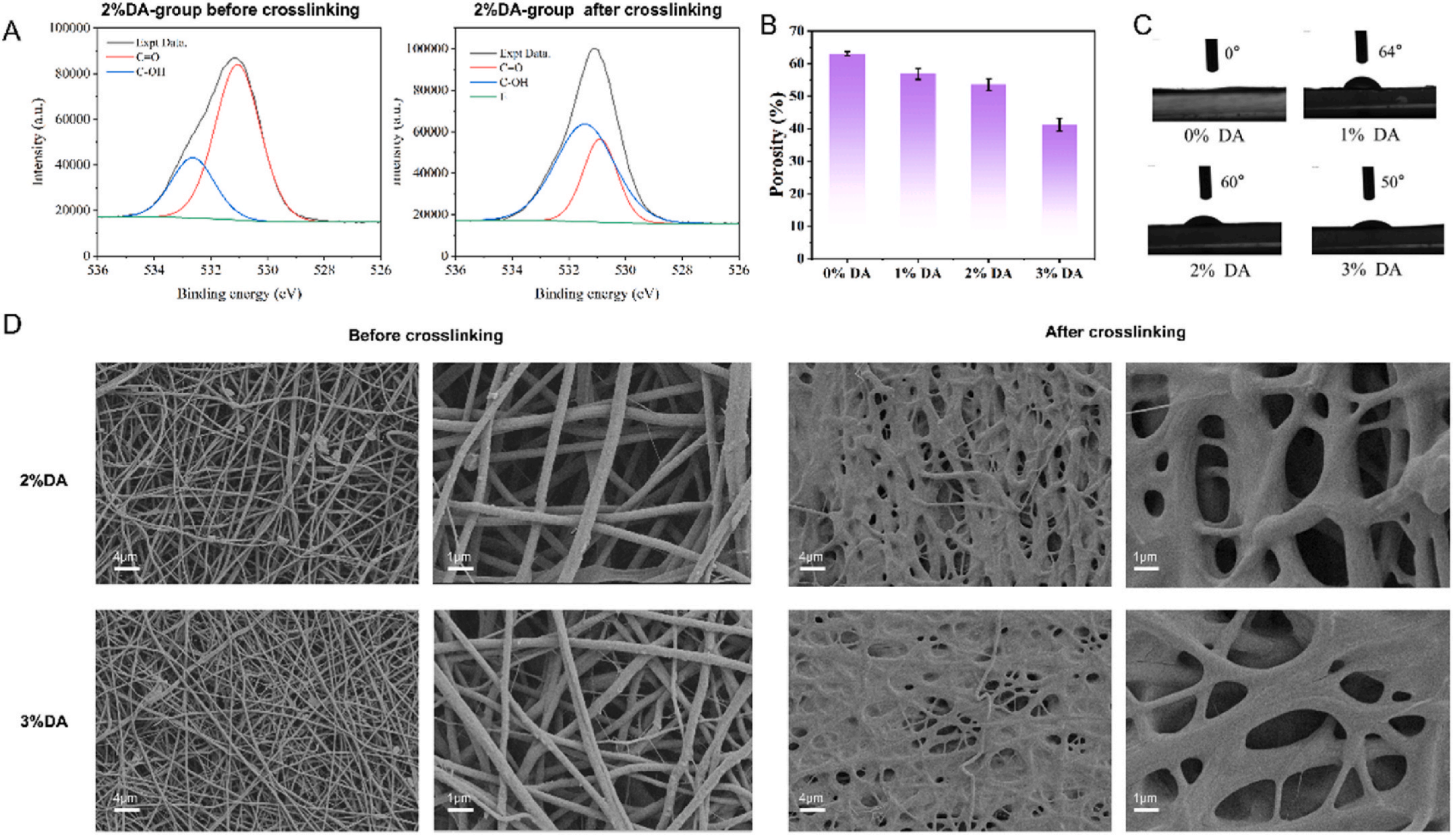
X-ray Photoelectron Spectroscopy (XPS) spectra of the 2% DA/PCL@GelMA before and after crosslinking. It revealed that DA was partially oxidized before crosslinking, and after crosslinking the C–O/C=O ratio increased, and the oxidation degree decreased (A). Porosity of 0%, 2% and 3% DA/PCL@GelMA after crosslinking (B). Water Contact Angle of 0%, 2% and 3% DA/PCL@GelMA after crosslinking (C). SEM images of 2% and 3% DA/PCL@GelMA, before and after crosslinking. SEM of 2%DA material was in Figs. 1 and 3%DA material was supplemented into this figure to demonstrate the difference in porosity between the two groups after crosslinking (D).

**Appendix. Table1:** Primer sequences corresponding to target genes used in qPCR experiments

Gene	Primer sequence (5′-3′)
*GAPDH F*	AGGTCGGTGTGAACGGATTTG
*GAPDH R*	TGTAGACCATGTAGTTGAGGTCA
*INOS F*	CTATCAGGAAGAAATGCAGGAGAT
*INOS R*	GAGCACGCTGAGTACCTCATTC
*IL1β F*	ACCCCAAAAGATGAAGGGCT
*IL1β R*	GATACTGCCTGCCTGAAGCTCT
*IL6 F*	AGTTGCCTTCTTGGGACTGA
*IL6 R*	TCCACGATTTCCCAGAGAAC
*Tnfα F*	TCTTCTCATTCCTGCTTGTGG
*Tnfα R*	GAGGCCATTTGGGACTTCT
*Arg1 F*	CCAGATGTACCAGGATTCTC
*Arg1 R*	AGCAGGTAGCTGAAGGTCTC
*IL10 F*	ACTCTTCACCTGCTCCACTG
*IL10 R*	GCTATGCTGCCTGCTCTTAC
*Tgfβ F*	CTTCAGCCTCCACAGAGAAGAACT
*Tgfβ R*	TGTGTCCAGGCTCCAAATATAG
*PPARγ F*	AACCCACAACCAAATCCACAC
*PPARγ R*	ATCACGGAGAGGTCCACAGA

## Data Availability

Data will be made available on request.

## Footnotes

Appendix A Supplementary data to this article can be found online at https://doi.org/10.1016/j.mtbio.2026.103272.
